# Impact of COVID-19 on the Lifestyle of Students of Taibah University, Madinah

**DOI:** 10.7759/cureus.43371

**Published:** 2023-08-12

**Authors:** Hanan Elsayed, Lujain Alrohaily, Saba L Alsaedi, Sulafah M Aljohani, Raghad A Jan, Nada N Alharthi, Reem A Garah

**Affiliations:** 1 Psychiatry, Mansoura University, Mansoura, EGY; 2 Psychiatry, Taibah University, Madinah, SAU; 3 College of Medicine, Taibah University, Madinah, SAU

**Keywords:** covid-19, sleep, diet, physical activity, lifestyle, lockdown

## Abstract

Background

The coronavirus disease 2019 (COVID-19) lockdown affected the daily habits of people around the world. Many countries have contributed to restricting its transmission by ensuing a quarantine, including Saudi Arabia, by shutting down educational facilities. Therefore, the system adapted to those changes, and people were required to stay home for work and education.

Objectives

This study aimed to explore the effect of the lockdown and house confinement during the COVID-19 pandemic on the important aspects of lifestyle, such as body weight, dietary habits, sleeping patterns, stress levels, screen time, and physical activity among students of Taibah University in Madinah, Saudi Arabia.

Methods

A cross-sectional study was carried out by a self-administered online questionnaire that was quoted from a Palestinian study. Then was translated from English to Arabic. In total, 528 Taibah University students were eligible to participate. It addressed food-related behavior (types of foods consumed) and lifestyle behavior (physical activity, sleep quality, and screen time).

Results

Study participants aged from 17 to 30 years, males and females. Mainly non-infected COVID-19 persons previously (54.4%). Participants showed an increase in body weight, intake of fried food, intake of sweets, sleep hours, screen time, and physical activity during lockdown. The most reported sources of stress during lockdown were staying at home all day (62.3%) and distance learning (44.9%).

Conclusion

The COVID-19 lockdown and the closure of universities have led to many changes in the everyday routine of university students, leading to changes in their lifestyle behaviors.

## Introduction

The coronavirus disease 2019 (COVID-19) started in Wuhan, Hubei, China, in December 2019 [1]. It is a viral infection that is caused by severe acute respiratory syndrome coronavirus-2 (SARS-CoV-2). COVID-19 began to spread through various countries, affecting people of all ages and ethnicities. Its spread made it a global threat, and soon after, it became a public health emergency. The first case ascertained of COVID-19 in Saudi Arabia was on March 3, 2020 [2]. Subsequently, on March 11, 2020, the World Health Organization (WHO) declared this a pandemic [3].

To prevent the further spread of the highly contagious virus, worldwide governments including Saudi Arabia implemented measures of social distancing and quarantine. Furthermore, the temporary suspension of Umrah travel restrictions and curfews. Citizens were completely banned from venturing outside and coming into contact with each other [4].

The lockdown was conducted on most public and private services, including schools and universities which lead to great consequences in lifestyle among young age groups. To overcome the learning disruption, distance learning was implanted. However, this could potentially have a harmful effect on both physical and mental health [5].

In Saudi, the house confinement period lasted for almost one year [6]. During such a long time period, many aspects of the population’s lives were affected immensely including their social lives, economic state, physical and mental health as well as their sleep pattern. Changes in diets could be predicted, which might give rise to more snaking and unhealthy consumption of food. In addition to that, boredom that might result from staying at home for a long period of time and absence from work and social activities might also make life more stressful [7]. Also, levels of physical activity are anticipated to decrease, due to the restrictions of outdoor time and the chance of participation in activities such as walking or shopping. Therefore, people were prone to a more dangerous sedentary lifestyle. Saudi Arabia has a family-oriented culture, which highly values family relationships and social gatherings. After the strict rules of social distancing and quarantine, it's expected that these changes might affect the population's psychological health [8].

The obscurity of the virus’s nature among the population’s understanding, in addition to the vagueness of the timeline in which they will have to continue the quarantine with their daily routines changed so drastically all of a sudden, they were expected to experience anxiety, stress, and feelings of fear [8]. To our knowledge, there is a lack of research on how the COVID-19 lockdown affected the lifestyle of the population, especially in the age of university students. However, most of the research focus is mainly on dietary changes and mental health [9-12].

To be ready for future lockdowns, there is a need for proper precautions regarding the lifestyle of the population. Therefore, the aim of the study was to shine light on the impending consequences of the quarantine on Taibah University students in Madinah regarding their lifestyle changes.

## Materials and methods

A cross-sectional study was conducted at Taibah University, Madinah, Saudi Arabia. The duration of data collection was three months, from February to the end of April 2022. The study protocol and questionnaire were revised by the Medical Research Ethics Committee, College of Medicine, Taibah University. Ethical approval was obtained on February 2022 (study ID: STU-21-028).

The study was carried out by a self-administered online questionnaire that was quoted from a Palestinian study by Allabadi et al. [13]. Then, was translated from English to Arabic and was reviewed by a psychiatrist who has expertise in this field. The questionnaire was randomly sent out on social media groups, such as WhatsApp and Telegram, to all Taibah University students. In addition to the socio-demographic data, the questionnaire focused on lifestyle habits including dietary habits, physical activity, screen time, sleep patterns, medical history, and sources of stress during lockdown.

In total, 528 Taibah University students were eligible to participate. In this study, male and female students from Taibah University were included. The exclusion criteria were composed of students with known chronic medical or psychological diseases that could impact their lifestyle. Additionally, students who did not complete the questionnaire and those who are taking medications such as isotretinoin and hormonal medications like oral contraceptive pills were excluded from the study.

A pilot study was done before the start of the research. The sample contained 20 students who were not included in the main study. Their ages ranged from 17 to 23 years and 85% of them were female. The construct validity was evaluated by item correlations with the total score on the questionnaire. Internal consistency between the questionnaire items was evaluated by counting the Cronbach alphas. The correlation coefficients of the questionnaire items ranged between 0.3 and 0.68. Most of the correlation coefficients were accepted and statistically significant. The internal consistency according to Cronbach's alpha coefficient was for the questionnaire items was 0.7, which considered an appropriate value for the purposes of this study.

Statical analysis

The SPSS version 26.0 (Chicago, IL: SPSS Inc.) was used for data analysis. Data normality was tested using the Kolmogorov-Smirnov test. Continuous data were presented as the median and interquartile range (IQR), while categorical variables were represented as frequency and percentages. McNemar’s test was used to compare the changes in physical activity between the two time periods (before lockdown and during lockdown). Also, changes in other lifestyle habits, such as the change in screen time were analyzed using the Wilcoxon signed-rank test. Categorical variable analysis was performed using the chi-square test, and Fisher’s exact test was used to determine if there was a significant association between weight change and lifestyle factors. Multinomial regression models were built to predict factors associated with weight changes. A p-value less than 0.05 was considered statistically significant, and the confidence interval was 95%.

## Results

The sample consisted of 528 students from Taibah University in Madinah. Table 1 illustrates the sociodemographic characteristics of the sample, 19.5% (n=103) reported their gender as male while 80.5% (n=425) as female. Most of the participants (88.4%) were at the age interval of 17-23 years. The median weight of the students was 55 kg (IQR=23), their median height was 159 cm (IQR=10), and 47.5% of students had normal weight. Approximately 33% of the students are in their first year of college. Also, 69.1% of the student reported that there was no change in the family's financial situation during the lockdown, as well 65.7% reported there was no change in the family's financial situation after the lockdown. Moreover, 54.4% of the sample did not get infected with COVID-19, compared to 35% of them who got infected with COVID-19.

**Table 1:** Socio-demographic characteristics of the studied students (n=528).

Characteristics		n (%)
Gender	Female	425 (80.5%)
	Male	103 (19.5%)
Age (years)	17-23	467 (88.4%)
	24-30	61 (11.6%)
Educational level (college year)	First year	175 (33.1%)
	Second year	40 (7.6%)
	Third year	101 (19.1%)
	Fourth year	90 (17.0%)
	Fifth year	64 (12.1%)
	Sixth year	58 (11.0%)
Weight (kg)	Median (IQR)	55 (17)
Height (cm)	Median (IQR)	159 (10)
BMI (kg/m^2^)	Median (IQR)	22 (5)
	Underweight	152 (28.8%)
	Normal weight	251 (47.5%)
	Overweight	97 (18.4%)
	Obese	28 (5.3%)
Family's financial situation during the lockdown	Better	99 (18.8%)
	Worse	32 (6.1%)
	No change	365 (69.1%)
	Unknown	32 (6.1%)
Family financial situation after the lockdown	Better	57 (10.8%)
	Worse	92 (17.4%)
	No change	347 (65.7%)
	Unknown	32 (6.1%)
Got infected with COVID-19	No	287 (54.4%)
	Yes	185 (35.0%)
	Unknown	56 (10.6%)

Table 2 shows the association between weight changes during the lockdown and socio-demographic characteristics of the sample, and lifestyle factors. Fisher's exact test shows that there was a statistically significant association between weight changes during lockdown and family financial situation during lockdown (p=0.018). Post hoc comparisons show that there were differences between students who had no change in their weight during the lockdown and unknown family financial situations during the lockdown. Also, Pearson's chi-square test shows that there was a statistically significant association between weight increase during the lockdown and getting infected with COVID-19 (p=0.013). Post hoc comparisons show there were differences for students who got not infected with COVID-19 compared to students who got infected with COVID-19. Furthermore, there was a statistically significant association between weight changes during the lockdown and food intake (p<0.001), and dietary habits during the lockdown, this relationship was positive with soft drinks intake (p=0.008), canned food intake (p<0.001), fried food intake (p=0.005), and sweets intake (p<0.001). Furthermore, Pearson's chi-square test shows that there was a statistically significant association between weight changes during the lockdown and doing exercises during the lockdown (p<0.001), there was a negative association between students who did exercises and had a decrease in their weight. As well as there was a statistically significant association between weight changes during lockdown and hours of sleep during lockdown (p=0.004), and there was a positive association between students who had an increase in their hours of sleep and an increase in their weight.

**Table 2:** Association between weight changes during the lockdown and socio-demographic characteristics and lifestyle habits of the sample. ^a^Fisher's exact test. ^b^Pearson's chi-square test. ^c^P-value<0.05 is statistically significant.

Factors	Categories	Weight changes during the lockdown				p-Value
		Increase	Decrease	No change	Unknown	
Gender^b^	Male	41 (7.8%)	23 (4.4%)	36 (6.8%)	3 (0.6%)	0.348
	Female	145 (27.5%)	112 (21.2%)	140 (26.5%)	28 (5.3%)	
Age^a ^(years)	17-23	158 (29.9%)	121 (22.9%)	160 (30.3%)	28 (5.3%)	0.333
	24-29	28 (5.3%)	14 (2.7%)	16 (3.0%)	3 (0.6%)	
Educational level^a^	First year	53 (10%)	44 (8.3%)	66 (12.5%)	12 (2.3%)	0.173
	Second year	7 (1.3%)	12 (2.3%)	18 (3.4%)	3 (0.6%)	
	Third year	39 (7.4%)	28 (5.3%)	28 (5.3%)	6 (1.1%)	
	Fourth year	33 (6.3%)	24 (4.5%)	29 (5.5%)	4 (0.8%)	
	Fifth year	23 (4.4%)	16 (3.0%)	20 (3.8%)	5 (0.9%)	
	Sixth year	31 (5.9%)	11 (2.1%)	15 (2.8%)	1 (0.2%)	
Family's financial situation during the lockdown^a^	Better	38 (7.2%)	33 (6.3%)	23 (4.4%)	5 (0.9%)	0.018^c^
	Worse	10 (1.9%)	7 (1.3%)	13 (2.5%)	2 (0.4%)	
	No change	130 (24.6%)	87 (16.5%)	131 (24.8%)	17 (3.2%)	
	Unknown	8 (1.5%)	8 (1.5%)	9 (1.7%)	7 (1.3%)	
Got infected with COVID-19^b^	No	85 (16.1%)	83 (15.7%)	99 (18.8%)	20 (3.8%)	0.013^c^
	Yes	77 (14.6%)	46 (8.7%)	54 (10.2%)	8 (1.5%)	
	Unknown	24 (4.5%)	6 (1.1%)	23 (4.4%)	3 (0.6%)	
Food intake during the lockdown^a^	Increase	149 (28.2%)	30 (5.7%)	61 (11.6%)	14 (2.7%)	<0.001^c^
	Decrease	8 (1.5%)	51 (9.7%)	13 (2.5%)	3 (0.6%)	
	No change	22 (4.2%)	49 (9.3%)	98 (18.6%)	12 (2.3%)	
	Unknown	7 (1.3%)	5 (0.9%)	4 (0.8%)	2 (0.4%)	
Did you do exercises during the lockdown^b^	No	114 (21.6%)	53 (10%)	116 (22%)	18 (3.4%)	<0.001^c^
	Yes	72 (13.6%)	82 (15.5%)	60 (11.4%)	13 (2.5%)	
Hours of sleep during the lockdown^a^	Increase	109 (20.6%)	57 (10.8%)	86 (16.3%)	11 (2.1%)	0.004^c^
	Decrease	13 (2.5%)	14 (2.7%)	8 (1.5%)	0 (0%)	
	No change	64 (12.1%)	64 (12.1)	82 (15.5%)	20 (3.8%)	
Suffered from insomnia during the lockdown^b^	No	138 (26.1%)	100 (18.9%)	139 (26.3%)	29 (5.5%)	0.084
	Yes	48 (9.1%)	35 (6.6%)	37 (7%)	2 (0.4%)	
Hours of television per week^a^	Less than 30 minutes	33 (6.8%)	32 (6.1%)	41 (7.8%)	9 (1.7%)	0.515
	1 to 2 hours	39 (7.4%)	37 (7%)	43 (8.1%)	5 (0.9%)	
	3 to 4 hours	43 (8.1%)	21 (4%)	32 (6.1%)	7 (1.3%)	
	5 hours or more	46 (8.7%)	25 (4.7%)	33 (6.8%)	4 (0.8%)	
	Unknown	25 (4.7%)	20 (3.8%)	27 (5.1%)	6 (1.1%)	
Hours of computers, tablets, per day^a^	Less than 30 minutes	2 (0.4%)	1 (0.2%)	1 (0.2%)	0 (0%)	0.086
	1 to 2 hours	8 (1.5%)	3 (0.6%)	7 (1.3%)	2 (0.4%)	
	3 to 4 hours	23 (4.4%)	25 (4.7%)	19 (3.6%)	0 (0%)	
	5 hours or more	149 (28.2%)	99 (18.8%)	139 (26.3%)	26 (4.9%)	
	Unknown	4 (0.8%)	7 (1.3%)	10 (1.9%)	3 (0.6%)	
Hours of school-related screen time per day^a^	Less than 30 minutes	7(1.3%)	9 (1.7%)	16 (3.0%)	2 (0.4%)	0.312
	1 to 2 hours	26 (4.9%)	17 (3.2%)	25 (4.7%)	5 (0.9%)	
	3 to 4 hours	60 (11.4%)	37 (7%)	44 (8.3%)	7 (1.3%)	
	5 hours or more	84 (15.9%)	67 (12.7%)	75 (14.2%)	13 (2.5%)	
	Unknown	9 (1.7%)	5 (0.9%)	16 (3.0%)	4 (0.8%)	
Change in water intake during the lockdown^a^	Increase	60 (11.4%)	48 (9.1%)	48 (9.1%)	10 (1.9%)	0.406
	Decrease	22 (4.2%)	15 (2.8%)	22 (4.2%)	4 (0.8%)	
	No change	84 (15.9%)	60 (11.4%)	91 (17.2%)	10 (1.9%)	
	Unknown	20 (3.8%)	12 (2.3%)	15 (2.8%)	7 (1.3%)	
Change in soft drinks intake during the lockdown^a^	Increase	53 (10%)	18 (3.4%)	28 (5.3%)	4 (0.8%)	0.008^c^
	Decrease	5 (9.5%)	50 (.5%)	63 (11.9%)	7 (1.3%)	
	No change	74 (14%)	60 (11.4%)	76 (14.4%)	15 (2.8%)	
	Unknown	9 (1.7%)	7 (1.3%)	9 (1.7%)	5 (0.9%)	
Change in vegetable intake during the lockdown^a^	Increase	58 (11%)	57 (10.8%)	55 10.4%)	8 (1.5%)	0.024^c^
	Decrease	23 (4.4%)	8 (1.5%)	11 (2.1%)	2 (0.4%)	
	No change	96 (18.2%)	65 (12.3%)	100 (18.9%)	15 (2.8%)	
	Unknown	9 (1.7%)	5 (0.09%)	10 (1.9%)	6 (1.1%)	
Change in fruit intake during the lockdown^a^	Increase	72 (13.6%)	52 (9.8%)	64 (12.1%)	8 (1.5%)	0.162
	Decrease	19 (3.6%)	11 (2.1%)	8 (1.5%)	4 (0.8%)	
	No change	89 (16.9%)	68 (12.9%)	96 (18.2%)	15 (2.8%)	
	Unknown	6 (1.1%)	4 (0.8%)	8 (1.5%)	4 (0.8%)	
Change in dairy intake during the lockdown^a^	Increase	78 (14.8%)	47 (8.9%)	51 (9.7%)	10 (1.9%)	0.005^c^
	Decrease	15 (2.8%)	15 (2.8%)	10 (1.9%)	1 (0.2%)	
	No change	90 (17%)	70 (13.3%)	105 (19.9%)	15 (2.8%)	
	Unknown	3 (0.6%)	3 (0.6%)	10 (1.9%)	6 (1.1%)	
Change in canned food intake during the lockdown^a^	Increase	62 (11.7%)	33 (6.3%)	30 (5.7%)	7 (1.3%)	<0.001^c^
	Decrease	27 (5.1%)	33 (6.3%)	29 (5.5%)	4 (0.8%)	
	No change	84 (15.9%)	63 (11.9%)	104 (19.7%)	12 (2.3%)	
	Unknown	13 (2.5%)	6 (1.1%)	13 (2.5%)	8 (1.5%)	
Change in fried food intake during the lockdown^a^	Increase	107 (20.3%)	48 (9.1%)	79 (15%)	15 (2.8%)	0.005^c^
	Decrease	23 (4.4%)	32 (6.1%)	22 (4.2%)	6 (1.1%)	
	No change	52 (9.8%)	50 (9.5%)	68 (12.9%)	8 (1.5%)	
	Unknown	4 (0.8%)	5 (0.9%)	7 (1.3%)	2 (0.4%)	
Change in sweets intake during the lockdown^a^	Increase	133 (25.2%)	60 (11.4%)	83 (15.7%)	15 (2.8%)	<0.001^c^
	Decrease	16 (3.0%)	37 (7.0%)	24 (4.5%)	4 (0.8%)	
	No change	36 (6.8%)	34 (6.4%)	64 (12.1%)	9 (1.9%)	
	Unknown	1 (0.2%)	4 (0.8%)	5 (0.9%)	3 (0.6%)	

The pattern of lifestyle habits of the sample was presented in Table 3. As noted, 35.2% of students gained weight during the lockdown. Food intake during lockdown increased by 48.1% of the sample, and the most cause of that increase was stress (43.4%). During the lockdown, the food mostly was homemade (94.1%). On the other hand, the hours of sleeping during lockdown increased among (49.8%) of the students, while 76.9% of the sample suffered from insomnia during the lockdown.

**Table 3:** Pattern of lifestyle habits of the studied students.

Characteristics		n (%)
Body weight during the lockdown	Increase	186 (35.2%)
	Decrease	135 (25.6%)
	No change	176 (33.3%)
	Unknown	31 (5.9%)
Food intake during the lockdown	Increase	254 (48.1%)
	Decrease	75 (14.2%)
	No change	181 (34.3%)
	Unknown	18 (3.4%)
Causes of increased food intake during the lockdown	Stress	229 (43.4%)
	Boredom	62 (11.7%)
	Pleasure	40 (7.6%)
During lockdown, on most days the food is	Homemade	497 (94.1%)
	Delivery	31 (5.9%)
Hours of sleep during the lockdown	Increased	263 (49.8%)
	Decreased	35 (6.6%)
	No change	210 (39.8%)
	Unknown	20 (3.8%)
Suffered from insomnia during the lockdown	Yes	406 (76.9%)
	No	122 (23.1%)

**Figure 1:**
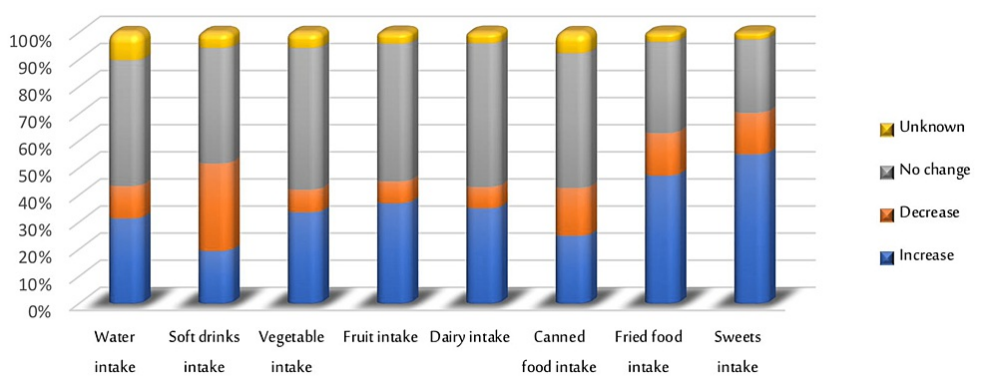
Change in dietary habits of the sample population during lockdown.

Figure 1 illustrates the change in dietary habits of the sample during the lockdown. A total of 46.4% of students reported there was no change in their water intake during the lockdown, likewise, 42.6% of them mentioned that there was no change in their intake of soft drinks. Also, the intake of vegetables, fruits, dairy, and canned food was not changed for most students during the lockdown. While intake of fried food and sweets increase by 47.2% and 55.1%, respectively, for most of the students during the lockdown.

Table 4 shows a comparison of the lifestyle habits of the students during and before lockdown. McNemar's test indicated that the median physical exercise of the students during lockdown was statistically significantly higher compared to before lockdown (p<0.001). Also, the Wilcoxon signed-rank test was used to compare screen time habits before and after the lockdown, and results show that the median hours of television during lockdown were statistically significantly higher compared to before the lockdown (p<0.001). And that the median hours of computers, tablets, and mobiles during lockdown were statistically significantly higher than before lockdown (p<0.001). Also, the median hours of school-related screen time during lockdown were statistically significantly higher than the median hours of school-related screen time before lockdown (p<0.001).

**Table 4:** Comparison of lifestyle habits (physical exercise, screen time) of the studied students during and before lockdown. ^a^McNemar's test. ^b^Wilcoxon signed-rank test. ^c^P-value<0.05 is statistically significant.

Characteristics		Before the lockdown, n (%)	During the lockdown, n (%)	p-Value
Physical exercise^a^	No	388 (73.5%)	301 (57.0%)	<0.001^c^
	Yes	140 (26.5%)	227 (43.0%)	
Hours of television per week^b^	Less than 30 minutes	219 (41.5%)	115 (21.8%)	<0.001^c^
	1 to 2 hours	104 (19.7%)	124 (23.5%)	
	3 to 4 hours	60 (11.4%)	103 (19.5%)	
	4 to 5 hours	41 (7.8%)	0 (0%)	
	5 hours or more	21 (4.0%)	108 (20.5%)	
	Unknown	83 (15.7%)	78 (14.8%)	
Hours of computers, tablets, and mobiles per day^b^	Less than 30 minutes	9 (1.7%)	4 (0.8%)	<0.001^c^
	1 to 2 hours	69 (13.1%)	20 (3.8%)	
	3 to 4 hours	204 (38.6%)	67 (12.7%)	
	5 hours or more	217 (41.1%)	413 (78.2%)	
	Unknown	29 (5.5%)	24 (4.5%)	
Hours of school-related screen time per day^b^	Less than 30 minutes	84 (15.9%)	346.4%)	<0.001^c^
	1 to 2 hours	199 (37.7%)	73 (13.8%)	
	3 to 4 hours	126 (23.9%)	148 (28.0%)	
	5 hours or more	87 (16.5%)	239 (45.3%)	
	Unknown	32 (6.1%)	34 (6.4%)	

**Figure 2:**
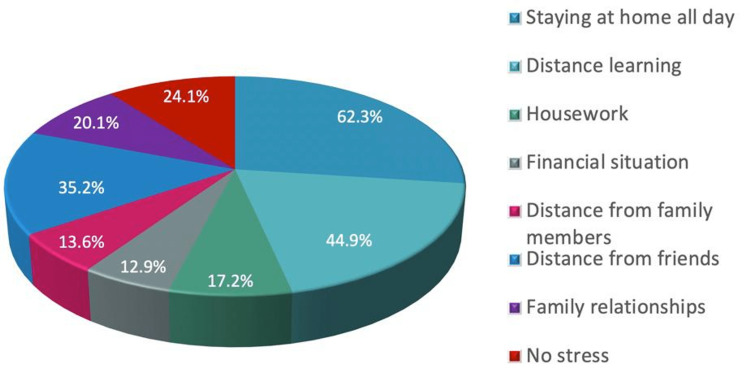
Sources of stress during lockdown among the studied students.

Figure 2 shows the sources of stress during lockdown among the students. A high ratio of students reported that "staying at home all day" was the source of stress for most (62.3%), followed by "distance learning" (44.9%). While "financial situation" was the source of stress for few. Also, about 24% of students reported they did not suffer from stress during the lockdown.

Table 2 shows the association between weight changes during the lockdown and socio-demographic characteristics of the sample, and lifestyle factors. Fisher's exact test shows that there was a statistically significant association between weight changes during lockdown and family financial situation during lockdown (p=0.018). Also, Pearson's chi-square test shows that there was a statistically significant positive association between weight increase during the lockdown and getting infected with COVID-19 (p=0.013). Furthermore, there was a statistically significant association between weight changes during the lockdown and food intake (p<0.001), and dietary habits during the lockdown, this relationship was positive with soft drinks intake (p=0.008), canned food intake (p<0.001), fried food intake (p=0.005), and sweets intake (p<0.001). Furthermore, Pearson's chi-square test shows that there was a statistically significant association between weight changes during the lockdown and doing exercises during the lockdown (p<0.001), there was a negative association between students who did exercises and had a decrease in their weight. As well as there was a statistically significant association between weight changes during lockdown and hours of sleep during lockdown (p=0.004), there was a positive association between students who had an increase in their hours of sleep and an increase in their weight.

Multiple logistic regression results summarized in Table 5 were performed to ascertain the predictability of weight changes during the lockdown. Considering the exp(β), an odds ratio equal to 1 shows no effect; an odds ratio greater than 1 shows the variable in question increases the odds of the outcome target level, and an odds ratio less than 1 indicates the variable in question decreases the odds of the outcome target level. Table 5 shows the p-values for family’s financial situation, changes in sleep pattern, and changes in dietary habits (soft drinks, vegetables, dairy, canned, and sweets intake) were statistically significant as predictors of weight changes during the lockdown.

**Table 5:** The multinomial regression analysis of weight changes during the lockdown among lifestyle factors of students. *P-value<0.05 is statistically significant.

Effect		Increase				Decrease				No change			
		p-Value	OR	95% CI		p-Value	OR	95% CI		p-Value	OR	95% CI	
				Lower	Upper			Lower	Upper			Lower	Upper
Family’s financial situation during the lockdown ("unknown" is reference group)	Better	0.007*	6.650	1.677	26.375	0.013*	5.775	1.448	23.032	0.071	3.578	0.898	14.255
	Worse	0.113	4.375	0.705	27.161	0.241	3.063	0.472	19.879	0.075	5.056	0.847	30.176
	No change	0.001*	6.691	2.154	20.783	0.010*	4.478	1.432	13.998	0.002*	5.993	1.976	18.176
Change in sleep pattern ("no change" is reference group)	Change	0.002*	3.466	1.564	7.679	.089	2.017	0.898	4.532	0.070	2.084	0.943	4.607
Change in soft drinks intake ("unknown" is reference group)	Increase	0.009*	7.361	1.655	32.748	0.147	3.214	0.663	15.578	0.079	3.889	0.856	17.677
	Decrease	0.045*	3.968	1.029	15.297	0.022*	5.102	1.266	20.562	0.019*	5.000	1.305	19.161
	No change	0.107	2.741	0.804	9.339	0.108	2.857	0.795	10.271	0.098	2.815	0.826	9.587
Change in vegetable intake ("unknown" is reference group)	Increase	0.015*	4.833	1.357	17.215	0.003*	8.550	2.111	34.624	0.027*	4.125	1.176	14.466
	Decrease	0.025*	7.667	1.298	45.289	0.115	4.800	0.682	33.798	0.197	3.300	0.537	20.266
	No change	0.015*	4.267	1.327	13.714	0.014*	5.200	1.399	19.328	0.018*	4.000	1.269	12.613
Change in dairy intake ("unknown" is reference group)	Increase	0.001*	13.000	2.690	62.831	0.011*	7.833	1.604	38.250	0.148	2.550	0.716	9.077
	Decrease	0.011*	25.00	2.09	298.3	0.011*	25.00	2.10	298.29	0.174	5.00	0.492	50.83
	No change	0.003*	10.00	2.16	46.28	0.009*	7.778	1.67	36.14	.041*	3.50	1.05	11.65
Change in canned food intake ("unknown" is reference group)	Increase	0.005*	5.451	1.679	17.693	0.007*	6.286	1.652	23.916	0.115	2.637	0.790	8.802
	Decrease	0.042*	4.154	1.055	16.355	0.002*	11.000	2.498	48.433	0.032*	4.462	1.137	17.504
	No change	0.007*	4.308	1.480	12.539	.002*	7.000	2.056	23.838	0.002*	5.333	1.840	15.460
Change in sweets intake ("unknown" is reference group)	Increase	0.006*	26.600	2.600	272.11	0.178	3.000	0.606	14.864	0.125	3.320	0.716	15.384
	Decrease	0.053	12.000	0.971	148.32	0.037*	6.938	1.126	42.731	0.158	3.600	0.607	21.352
	No change	0.041*	12.000	1.113	129.42	0.221	2.833	0.535	15.014	0.074	4.267	0.868	20.972

## Discussion

The COVID-19 lockdown affected the daily habits of people around the world. Many countries have contributed to restricting its transmission by ensuing a quarantine, including Saudi Arabia, by shutting down educational facilities. Therefore, the system adapted to those changes, and people were required to stay home for work and study.

Presently, there is a gap in knowledge about the effects of the COVID-19 lockdown on lifestyle habits among university students. To our knowledge, this is the first study to examine how the lockdown impacted this target population's lifestyle habits in Madinah, Saudi Arabia. The current study’s findings indicate that some changes have occurred in the lifestyle behavior of college students during lockdown. There was an increase in body weight, intake of fried food, intake of sweets, sleep hours, screen time, and physical activity during the lockdown. The most reported sources of stress during lockdown were staying at home all day (62.3%) and distance learning (44.9%).

This study’s participants exhibited an increase in weight that can be explained by the type of food intake, such as an increase in consumption of fried food and sweets, and changes in lifestyle during the lockdown. In concordance with the study of Allabadi et al., which was conducted on adolescent students (n=600) in Palestine, and a study by Bakhsh et al., a cross-sectional Saudi study (n=2255), both showed similar findings in food intake, weight gain, increase screen time and sleep hours [13,14]. On the other hand, a study by Braiji et al., which was conducted in Jeddah on Saudi citizens (n=476), aged between 20 and 60 years showed that many participants lost weight [8]. This may be explained by the fact that older people preferred to eat healthy foods to strengthen their immune systems and avoid getting infected with COVID-19. As all students attended their classes on computers, phones, or tablets, the increase in screen time found in this study was expected because of sitting at home all day and online teaching.

Unexpectedly, an increase in physical exercise during lockdown was found in this study’s population compared to before, as there was an increase from 26.5% to 43%. This increase might be explained by the daily walking permeation by the Saudi government for 60 minutes within 1 km of the residence [15]. This permeation could have been the only possible source of ventilation and change of scenery those students had. Which could have been a substitute for physical exercise. Furthermore, the majority of the participants, amounting to approximately 57%, didn’t do physical exercise during lockdown. This could explain the contradictory increase in weight found in this study. In the study by Alhusseini et al., conducted in Saudi Arabia on adults aged 18 years and more (n=1051), similar results were confirmed [16].

The results of the current study further highlight the strong impact of the COVID-19 lockdown on dietary habits, which leads to increased intake of homemade food (94.1%), especially easier recipes, such as sweets and fried foods, in comparison to delivered food (5.9%). This can be explained by longer hours of staying at home. Furthermore, the fear of delivered food being contaminated with COVID-19. In the same line as Bakhsh et al.'s study and a study conducted in Denmark by Giacalone et al. on citizens aged more than 18 years (n=2,462) showed that there was an increase in homemade food, especially pastries, and a lower rate of ordering fast food [14,17].

Generally, the lockdown had a negative effect on eating habits. More food was being consumed throughout the home confinement, which could be attributed to feelings of boredom and stress. In addition, due to grocery shopping restrictions, people engaged in food stocking increased the availability and amount of food to be consumed in the house.

In a similar line to the study by Alhusseini et al., sleeping hours have also increased significantly during the lockdown in contrast to the past-quarantine time [16]. This was reported by 49.8% of responders. Despite this, the quality of sleep has been negatively affected in 76.9% who reported experiencing insomnia and poor sleep quality. This could be associated with the increase in the variety of stressors during lockdown most of which were staying at home all day with nothing to do and distance learning causing emotional exhaustion.

The results of this study’s regression analysis identified the family’s financial situation during lockdown, changes in sleep pattern during lockdown, and changes in dietary habits during lockdown (soft drinks, vegetables, dairy, canned, and sweets intake) as significant predictors of weight changes during the lockdown (p<0.05).

Some potential limitations in the present study included the liability for subjective overestimation and underestimation of some factors such as body weight changes and physical activity. Also, despite distributing the survey to both genders, more females have answered the survey. Therefore, it led to an unequal gender presentation, which might limit the generalization of the results. In addition, we have excluded patients with medical and psychological illnesses. This could further limit the generalization of this study and create some bias.

## Conclusions

In conclusion, this study has revealed the effect of the COVID-19 lockdown and the closure of universities and how they have led to many changes in the everyday routine of university students, leading to changes in their lifestyle behaviors. The current study found an increase in many unfavorable habits, such as fried food and sweets intake, increased screen time, and sleeping hours. These bad eating and lifestyle choices may contribute to more overweight/obesity problems and perhaps, later on, the burden of chronic diseases. Further studies are still needed to explore the effect of lockdowns on lifestyle behavior, also, to study the effects of the lockdown on other age groups and in different populations, and evaluate the possible role of culture. Moreover, studies are recommended to study the lifestyle in the era after COVID-19 to determine whether the observed lifestyle changes are still present with the continuity of some of the associated factors, such as online learning. Systematized efforts are recommended to increase the awareness of the population on how to accommodate during stressful times.

## References

[1] Chan JF, Yuan S, Kok KH (2020). A familial cluster of pneumonia associated with the 2019 novel coronavirus indicating person-to-person transmission: a study of a family cluster. Lancet.

[2] Hassounah M, Raheel H, Alhefzi M (2020). Digital response during the COVID-19 pandemic in Saudi Arabia. J Med Internet Res.

[3] Cucinotta D, Vanelli M (2020). WHO declares COVID-19 a pandemic. Acta Biomed.

[4] (2023). Saudi Arabia bans prayers at mosques over coronavirus fears. https://www.aljazeera.com/news/2020/3/20/saudi-arabia-bans-prayers-at-mosques-over-coronavirus-fears.

[5] Banerjee D, Rai M (2020). Social isolation in COVID-19: the impact of loneliness. Int J Soc Psychiatry.

[6] (2023). Saudi Arabia lifts COVID-19 precautionary measures. https://www.moh.gov.sa/en/Ministry/MediaCenter/News/Pages/News-2022-06-16-001.aspx.

[7] Al-Saleh MM, Alamri AM, Alhefzi AA, Assiri KK, Moshebah AY (2021). Population healthy lifestyle changes in Abha city during COVID-19 lockdown, Saudi Arabia. J Family Med Prim Care.

[8] Braiji EH, Abduljawad EA, Alrasheedi AA (2022). Impact of COVID-19 pandemic quarantine on dietary behaviors and lifestyle of Saudi adults in Jeddah, Kingdom of Saudi Arabia. Saudi Med J.

[9] Alfawaz H, Yakout SM, Wani K (2021). Dietary intake and mental health among Saudi Adults during COVID-19 lockdown. Int J Environ Res Public Health.

[10] Abd El-Fatah NK, Alshehri AA, Alsulami FH, Alasmari N, Osman NA (2021). Association between mental health outcomes and changes in lifestyle behavior index among Saudi adults 16 weeks after COVID-19 pandemic lockdown release. Front Public Health.

[11] Alfawaz H, Amer OE, Aljumah AA (2021). Effects of home quarantine during COVID-19 lockdown on physical activity and dietary habits of adults in Saudi Arabia. Sci Rep.

[12] Hanbazaza M, Wazzan H (2021). Changes in eating habits and lifestyle during COVID-19 curfew in children in Saudi Arabia. Nutr Res Pract.

[13] Allabadi H, Dabis J, Aghabekian V, Khader A, Khammash U (2020). Impact of COVID-19 lockdown on dietary and lifestyle behaviours among adolescents in Palestine. DHH.

[14] Bakhsh MA, Khawandanah J, Naaman RK, Alashmali S (2021). The impact of COVID-19 quarantine on dietary habits and physical activity in Saudi Arabia: a cross-sectional study. BMC Public Health.

[15] (2023). Saudi Arabia allows 1-hour walk amid coronavirus lockdown. https://saudigazette.com.sa/article/593491.

[16] Alhusseini N, Alammari D, Ramadan M (2022). The impact of COVID-19 pandemic on lifestyle among the Saudi population. J Public Health Res.

[17] Giacalone D, Frøst MB, Rodríguez-Pérez C (2020). Reported changes in dietary habits during the COVID-19 lockdown in the Danish population: the Danish COVIDiet Study. Front Nutr.

